# Supplementary material for: An integrated blockchain and IPFS-based solution for secure and efficient source code repository hosting using middleman approach
Source: PLoS One. 2025 Sep 3;20(9):e0331131. doi: 10.1371/journal.pone.0331131 (PMC12407423; doi:10.1371/journal.pone.0331131)
Supplement: S1 Appendix — This appendix contains the detailed, trial-by-trial results and summary tables from the performance evaluation for the public testnet trials. (PDF) [file pone.0331131.s001.pdf]

Supporting Information

S1 Appendix: Detailed Experimental Data

This appendix contains the detailed, trial-by-trial results and summary tables from the performance evaluation conducted for the manuscript, "An Integrated Blockchain and IPFS-based Solution for Secure and Efficient Source Code Repository Hosting using Middleman Approach."

Table 1: Raw Experimental Data for Public Testnet Trials (Latency in ms)

File Size	Trial	Encrypt	IPFS Upload	BC Confirm	Gas Used	Pull (Pre-Conf)	Pull (Post-Conf)
**1 MB**	1	99.40	2229.50	8859.60	206886	1267.00	1462.90
	2	105.40	1665.50	16931.30	206886	1284.70	1646.10
	3	106.50	1853.10	12860.90	206886	1324.60	1656.90
	4	103.80	1708.40	13312.40	206886	1251.80	1285.30
	5	104.40	2235.00	16904.40	206886	1298.40	2678.80
**5 MB**	1	393.00	6125.30	17384.00	206886	1949.40	2162.30
	2	412.80	3184.40	13375.50	206886	1914.90	1904.70
	3	388.00	3437.90	13375.90	206886	4191.00	2150.90
	4	409.10	2903.10	8972.60	206886	2011.00	1932.40
	5	411.90	3290.00	8988.00	206886	1971.60	2226.90
**10 MB**	1	769.40	8135.70	16994.80	206886	2672.30	3003.20
	2	802.20	4587.70	4985.60	206886	2570.50	2814.90
	3	800.90	5465.40	12946.70	206886	2465.80	2998.60
	4	802.50	5060.60	8871.40	206886	2482.40	2526.20
	5	824.20	5566.80	16969.80	206886	2484.50	2827.50
**20 MB**	1	1579.70	11364.50	13200.40	206886	3967.40	5389.40
	2	1526.00	8860.70	17197.90	206886	4454.50	3858.20
	3	1563.80	8883.90	21509.30	206886	3939.50	3954.80
	4	1658.90	11963.40	4906.80	206886	3877.60	3974.80
	5	1582.50	8389.80	9374.40	206886	4151.50	3789.50

Table 2: Summary of Performance Metrics (Average ± Standard Deviation)

File Size	Metric	Value (ms)	Value (s)
**1 MB**	Encryption Latency	103.9 ± 4.5	0.10 ± 0.00
	IPFS Upload Latency	1938.3 ± 323.2	1.94 ± 0.32
	Blockchain Latency	15593.7 ± 3058.4	15.59 ± 3.06
	Pull Latency (Pre-Conf)	1285.3 ± 33.7	1.29 ± 0.03
	Pull Latency (Post-Conf)	1746.0 ± 541.6	1.75 ± 0.54
**5 MB**	Encryption Latency	403.0 ± 11.2	0.40 ± 0.01
	IPFS Upload Latency	3788.1 ± 1289.4	3.79 ± 1.29
	Blockchain Latency	12519.2 ± 3349.5	12.52 ± 3.35
	Pull Latency (Pre-Conf)	2407.6 ± 1010.5	2.41 ± 1.01
	Pull Latency (Post-Conf)	2075.0 ± 131.5	2.08 ± 0.13
**10 MB**	Encryption Latency	799.8 ± 18.9	0.80 ± 0.02
	IPFS Upload Latency	5763.2 ± 1393.7	5.76 ± 1.39
	Blockchain Latency	12153.7 ± 4771.5	12.15 ± 4.77
	Pull Latency (Pre-Conf)	2535.1 ± 85.8	2.54 ± 0.09
	Pull Latency (Post-Conf)	2834.1 ± 191.0	2.83 ± 0.19
**20 MB**	Encryption Latency	1582.2 ± 48.6	1.58 ± 0.05
	IPFS Upload Latency	9892.5 ± 1530.8	9.89 ± 1.53
	Blockchain Latency	13237.8 ± 6195.6	13.24 ± 6.20
	Pull Latency (Pre-Conf)	4078.1 ± 210.0	4.08 ± 0.21
	Pull Latency (Post-Conf)	4193.3 ± 658.2	4.19 ± 0.66
