# Supplementary material for: An integrated blockchain and IPFS-based solution for secure and efficient source code repository hosting using middleman approach
Source: PLoS One. 2025 Sep 3;20(9):e0331131. doi: 10.1371/journal.pone.0331131 (PMC12407423; doi:10.1371/journal.pone.0331131)
Supplement: Attachment — Submitted filename: response_to_reviewer.pdf [file pone.0331131.s002.pdf]

July 2, 2025

To:
Dr. Yang (Jack) Lu, Academic Editor,
And to the Esteemed Reviewers,
PLOS ONE

**Subject: Response to Reviewers for Manuscript PONE-D-24-43266**
**Title:** "An Integrated Blockchain and IPFS-based Solution for Secure and Efficient Source Code Repository Hosting using Middleman Approach"

Dear Dr. Lu and Esteemed Reviewers,

Thank you for your time and for providing such insightful and constructive feedback on our manuscript, "An Integrated Blockchain and IPFS-based Solution for Secure and Efficient Source Code Repository Hosting using Middleman Approach" (PONE-D-24-43266). We are grateful for the opportunity to revise our work. We agree with the reviewers that the original submission had significant room for improvement, particularly concerning the need for empirical validation and clearer articulation of the system's novelty.

We have undertaken a comprehensive revision to address every point raised. The most significant change is the inclusion of a full-scale empirical evaluation. We implemented a complete prototype of our proposed architecture, secured it with Shamir's Secret Sharing (SSS), and deployed it to the public Sepolia testnet. This has allowed us to validate our theoretical discussions with quantitative, data-driven analysis of the system's performance, security, and novelty.

**Please note:** Given the extensive nature of these revisions, which include a full empirical study and the rewriting of major sections as requested, a standard line-by-line track-changes document was found to be impractical. To better guide your review, we have instead highlighted all new or significantly rewritten paragraphs and sections in blue text in the 'Revised Manuscript with Track Changes' file. Minor typographical and grammatical corrections have not been highlighted for clarity.

We believe the manuscript is now substantially stronger and makes a more significant contribution to the field. Below, we provide a point-by-point response to the comments.

---

## Response to Reviewer #1

*Comment 1: "Abstract and Conclusion must be revised. Make is concise but solid."*

**Response:** We thank the reviewer for this valuable suggestion. We agree that the original versions were too descriptive. We have completely rewritten the Abstract and Conclusion to be more concise and to reflect the empirical findings of our study. The new versions are data-driven and confidently state the achievements of our work. We have completely rewritten the Abstract and Conclusion to be more concise and to reflect the empirical findings of our study.

*Comment 2: "It is suggested to avoid writing short paragraphs which cause inconvenience in writing flow."*

**Response:** We appreciate this stylistic advice. We have carefully reviewed the entire manuscript and have merged short paragraphs, particularly in the 'Related Works' and 'Introduction' sections, to improve the overall narrative flow.

*Comment 3: "Motivation and Organization of this study must be clear defined."*

**Response:** We thank the reviewer for this important point. We have added a new, explicit paragraph at the end of the 'Introduction' section that clearly outlines the primary motivations for our work and details the organization of the paper, guiding the reader through the manuscript's structure.

*Comment 4: "Figures must be revised and an interested presentation should be adopted with high resolution quality. If possible, check and revise figure captions, they are too long."*

**Response:** We have taken this feedback to heart. We have revised Fig1 and Fig2 to increase clarity and generated a new set of high-resolution charts based on our experimental data (see new Figures 3, 4, and 5 in the revised manuscript). We have also revised all figure captions throughout the manuscript to be more concise and impactful while still accurately describing the content, as seen in these figures.

*Comment 5: "The authors should carefully check if all abbreviations are defined at the first place for better readability."*

**Response:** We have performed a thorough check of the entire manuscript and have now defined all technical abbreviations (e.g., VCS, IPFS, SSS, IIoT, IoV, NFV) upon their first use to enhance readability.

*Comment 6 & 7: "Reference literature must be updated and suggested to add following articles... Material and Methods are fine. Better to improve writing for results discussion."*

**Response:** We thank the reviewer for these literature suggestions. We have carefully reviewed the recommended articles and have now integrated them into our revised 'Related Works' section to better contextualize our research. Furthermore, the 'Results and Discussion' section has been completely rewritten from the ground up, based on our new empirical data, which we believe substantially improves the quality of the discussion.

## Response to Reviewer #2

*Comment 1: Lack of Empirical Evaluation: "The manuscript fails to provide quantitative performance results. Any benchmark, latency analysis, or transaction cost metrics do not support claims regarding scalability, efficiency, and performance improvement over existing systems. Provide experimental evaluations comparing Blockchain gas fees with/without Middleman IPFS, upload/download time and throughput metrics, and comparisons with traditional centralized systems (e.g., GitHub/GitLab)."*

**Response:** We thank Reviewer #2 for this critical and essential feedback. We agree completely that the original manuscript's claims required robust empirical validation. To address this, we have implemented a full prototype of our proposed architecture and conducted a comprehensive performance evaluation on the public Sepolia testnet.

The new 'Results and Discussion' section (Section 4) is now rewritten completely to presenting this data. Specifically:

- We provide a direct performance comparison against a centralized Git/GitHub baseline in Table 1 and Figure 3, analyzing both push and pull operations.
- We present a detailed breakdown of system latency to identify bottlenecks in Figure 4.
- We report the consistent on-chain gas costs for repository registration in the 'System Overhead and Bottleneck Analysis' subsection.

We believe this new data fully addresses the reviewer's concern and substantiates the performance claims of our system with rigorous, quantitative evidence.

**In accordance with PLOS ONE's data availability policy, all underlying data for our findings are provided in the Supporting Information files. Furthermore, the complete source code for our prototype and middleware has been made publicly available on GitHub and permanently archived with a DOI via Zenodo (DOI: 10.5281/zenodo.15700465).**

*Comment 2: Unclear Novelty Beyond Engineering Integration: "The "Middleman IPFS" is essentially a cache or temporary node. While practical, this seems like an engineering optimization, not a fundamentally new algorithm or protocol. Clarify the scientific novelty of this middleware. Does it offer any guarantees (e.g., consistency, eventual deletion enforcement, reduced consensus cost)? If not, frame the contribution more as a design integration and prototype than a fundamental system innovation."*

**Response:** We thank the reviewer for this ciritical suggestion to clarify the novelty of our contribution. The reviewer correctly noted that a simple cache would be an engineering optimization. In our revised manuscript, we now clarify that the novelty lies not merely in the existence of the middleman, but in the **secure, high-performance protocol** it enables. We have strengthened this argument in two key ways:

1. **Security Protocol Novelty:** We have integrated **Shamir's Secret Sharing (SSS)** as a core component. As detailed in the new 'Security and Trust Model' subsection (Section 4.4), this renders the middleman cryptographically powerless and distributes trust, elevating the design beyond a simple cache to a trust-minimized protocol.
2. **Application-Layer Protocol Novelty:** We formally define our **'Authoritative-First, Optimistic-Fallback'** retrieval protocol. We argue in the revised 'Related Works' and 'Discussion' sections that this protocol is a novel contribution to solving the well-known problem of user-perceived latency in dApps, and our empirical data proves its effectiveness.

*Comment 3: Security and Trust Assumptions Underexplored: "The system assumes that the Middleman IPFS deletes keys post-confirmation, but does not specify how this is enforced or audited. Explain how deletion is verified. Consider potential attacks (e.g., replay, key retention, collusion). Add a formal threat model or table of assumptions."*

**Response:** We thank the reviewer for this important point. We have added a new, dedicated **'Security and Trust Model' subsection (Section 4.4)** to formally address this. We present a threat model and explain how the integration of SSS directly mitigates threats such as key retention by the middleman. We argue that, since the middleman holds only a single, insufficient key share, the security of the system does not depend on a trusted deletion mechanism. Furthermore, we clarify that the authoritative share is stored immutably on the blockchain, providing a permanent disaster recovery path.

*Comment 4: Lack of Usability and Developer Workflow Analysis: "The paper lacks consideration for developer experience. There's no discussion of CI/CD pipeline compatibility. Branching, merging, or pull request equivalents. Real-time editing conflicts and resolution. Add a section comparing the proposed system with Git-based developer workflows, especially in collaborative scenarios."*

**Response:** This is an excellent point. To address it, we have added a new subsection titled **'Comparative Analysis with Centralized VCS' (Section 4.1.1)**. This subsection uses our new experimental data (Table 1 and Figure 3) to provide a direct, data-driven comparison of the user-perceived latency of our system's 'push' and 'pull' operations against the equivalent commands in Git. This allows for a concrete discussion of the practical trade-offs for a developer.

*Comment 5: Overuse of Passive Language and Redundancy. Several sections are highly repetitive, particularly those that describe IPFS and blockchain properties. Condense redundant text. Emphasize what was implemented and tested, and reduce generic blockchain/IPFS descriptions already covered in existing literature.*

**Response:** We thank the reviewer for this stylistic feedback. We have thoroughly revised the entire manuscript to use more active and confident language, particularly in the Abstract, Introduction, and Conclusion. We have also condensed the 'Related Works' section, removing redundant descriptions of general blockchain/IPFS concepts to focus more on literature that directly informs or contrasts with our specific contributions.

---

We are confident that these substantial revisions have addressed all the concerns raised and have resulted in a much-improved manuscript. We once again thank the editor and the reviewers for their time and valuable contributions to our work.

Sincerely,

Sabbir Ahmed (on behalf of all authors)
Department of Computer Science and Engineering
Islamic University of Technology (IUT)
