# Supplementary material for: An integrated blockchain and IPFS-based solution for secure and efficient source code repository hosting using middleman approach
Source: PLoS One. 2025 Sep 3;20(9):e0331131. doi: 10.1371/journal.pone.0331131 (PMC12407423; doi:10.1371/journal.pone.0331131)
Supplement: Attachment — Submitted filename: R2_response_to_reviewer.pdf [file pone.0331131.s003.pdf]

August 7, 2025

To:
Dr. Yang (Jack) Lu, Academic Editor,
And to the Esteemed Reviewers,
PLOS ONE

Subject: Response to Reviewers for Manuscript PONE-D-24-43266R1

Dear Dr. Lu and Esteemed Reviewers,

Thank you for the further review of our manuscript and for the opportunity to submit a second revision. We are very grateful for the encouraging feedback and the insightful suggestions for improvement.

We have carefully considered all new comments and have revised the manuscript accordingly. We note that the feedback appears to come from two valuable perspectives, with Reviewer #3 providing forward-looking suggestions on our revised manuscript (R1), and Reviewer #4 providing a thorough analysis that aligns with the critiques of our original submission. We have structured our response below to address both sets of feedback, and we believe the manuscript is now significantly stronger as a result.

Response to Reviewer #3 (Dr. Chengyang Nie)

We thank Dr. Nie for his positive assessment and excellent suggestions for extending the discussion.

Comment 1: "...it is better that the manuscript could add a brief discussion about how the proposed system integrates with existing Ci/CD pipelines."

Response: This is an excellent point. We have now added a new paragraph in the 'Developer Workflow and Usability' subsection discussing exactly this. We outline how a command-line interface could wrap our system's core functions to enable integration into automated CI/CD pipelines for verifiable, decentralized builds and deployments.

Comment 2: "The conclusion mentions that the middleware could be evolved into a Dao-based service. I think it would be better that some details could be added..."

Response: Thank you for this critical observation. Our initial mention was too brief. We have expanded the 'Conclusion' section to include more specific design considerations for a future DAO implementation, such as managing a treasury for IPFS pinning and using token-based voting for governance.

Comment 3: "I think some figure captions could be further tightened."

Response: We thank the reviewer for this advice. We have reviewed and edited all figure captions throughout the manuscript to make them more concise while retaining their descriptive accuracy.

Response to Reviewer #4

We thank Reviewer #4 for their thorough and insightful review. The weaknesses identified by the reviewer regarding the lack of empirical data were perfectly aligned with the feedback we received in the first round. We are pleased to confirm that addressing these points was the primary focus of our first revision (R1), and the current manuscript now contains the comprehensive evaluation the reviewer has called for.

Weakness 1: Limitation in empirical analysis: "...the lack of empirical evaluation limits the paper's contribution. The authors should at minimum conduct simulation-based performance tests or benchmarking against centralized platforms..."

Response: We agree wholeheartedly. The current version of the manuscript is now built upon a comprehensive empirical evaluation. As detailed in the 'Results and Discussion' section, we have implemented a full prototype, deployed it to the public Sepolia testnet, and benchmarked its performance directly against a centralized Git/GitHub baseline across a range of file sizes.

Weakness 2: Performance Evaluation: "The evaluation is rather limited... Compare performance with centralized systems (e.g., GitHub); Include stress tests... Provide data on blockchain transaction throughput..."

Response: We have addressed these points directly in our 'Results and Discussion' section. To guide the reviewer to these specific additions, we have highlighted the key introductory sentences for each piece of evidence in blue text. in the 'Revised Manuscript with Track Changes' file:

- **Comparison with GitHub:** Our direct, quantitative comparison of 'push' and 'pull' latency against Git is introduced by the highlighted text in the 'Comparative Analysis with Centralized VCS' subsection and presented in Table 1 and Fig 3.
- **Stress Tests:** We conducted stress tests by scaling the file size from 1MB to 20MB. Fig 4 and the highlighted texts in the 'System Overhead and Bottleneck Analysis' subsection provides a detailed breakdown of where the system latency is spent under this load.
- **Blockchain Data:** The highlighted text in the same subsection report the consistent 'Gas Used' for on-chain transactions and provide a detailed analysis of the 'Pure Blockchain Latency', which is a more direct measure of the on-chain performance bottleneck than raw TPS for our use case.

We are confident that our new, data-driven 'Results and Discussion' section now provides the robust empirical evidence the reviewer correctly identified as a critical requirement.

We believe these latest revisions, combined with the major improvements from the first round, have resulted in a much stronger and more complete manuscript. We thank you again for your time and consideration.

Sincerely,

Sabbir Ahmed (on behalf of all authors)
